# Relationship between C2 slope with sagittal parameters and clinical function of degenerative cervical kyphosis

**DOI:** 10.1186/s13018-023-04011-0

**Published:** 2023-07-20

**Authors:** Zihao Chai, Xiaolu Yang, Haitao Lu, Yunlei Zhai, Wei Zhang, Haiyang Yu

**Affiliations:** 1 Department of Orthopedics, Fuyang People’s Hospital of Anhui Medical University, 501 Sanqing Road, Fuyang, 236000 Anhui China; 2 Spinal Deformity Clinical Medicine and Research Center of Anhui Province, 501 Sanqing Road, Fuyang, 236000 Anhui China

**Keywords:** Degenerative cervical kyphosis, C2 slope, Sagittal alignment, Health-related quality of life

## Abstract

**Purpose:**

To explore the relationship between C2 slope with sagittal parameters and clinical function of degenerative cervical kyphosis (DCK).

**Methods:**

A retrospective analysis of 127 patients with degenerative cervical spondylosis treated in our spinal deformity center from January 2019 to June 2022. Patients were categorized into two groups and compared based on C2-7 angle (C2-7 ≥ 5° as kyphosis group, C2-7 < 5° as lordosis group). Pearson correlation or Spearman correlation was used to analyze the relationship between C2S and conventional radiological parameters and health -related quality-of-life (HRQOL) outcomes as measured by the EuroQol 5 dimension questionnaire (EQ5D), NRS, and the neck disability index (NDI). The cutoff value of C2S was determined by a receiver operating characteristic (ROC) curve.

**Results:**

There were 127 patients who met inclusion criteria (79 men and 48 women). Average 56.00 ± 10.27 years old (range 31–81 years old). C2S of kyphosis group is higher than non-kyphosis group. Aggravating cervical kyphosis increases cSVA positively. For all patients, C2S demonstrated a significant correlation with the O-C2 angle, C2-7 angle, cSVA, and TS-CL (*p* < 0.05). NRS, NDI and EQ5D-VAS scores revealed a significant correlation with C2S and cSVA (*p* < 0.01). For the subgroup of patients presenting with DCK, ROC curves demonstrated the cutoff values of C2S as 26.3°, and 30.5°, according to a cSVA of 40 mm, and severe disability expressed by NDI, respectively.

**Conclusion:**

On the basis of retaining the consistency of cranio-cervical and cervico-thoracic structure, C2S can better analyze the sagittal alignment of DCK patients than TS-CL and has good practicability in clinical application and HRQOL evaluation.

## Introductions

As a cascade structural unit to coordinate the sagittal balance of the spine, cervical spine has important clinical significance in supporting the skull and maintaining functional vision [1]. With the change of behavior in modern society, human beings have changed from long-term stoop work to frequently bending of their neck during tasks, leading to a significant increase in the prevalence of cervical degenerative diseases, and some patients may appear cervical kyphosis [2]. In addition to axial symptoms, spinal cord and neurological function may be involved, and even the overall spinal mechanics may change accordingly. When the cervical vertebral sagittal position shows load-induced decompensation, the body is usually regulated by spinal “hinged” conduction to maintain balance and stability [3].

Cervical spine evaluation parameters commonly used in clinical practice include O-C2 angle, C2-C7 angle, cSVA, T1S, T1S-CL. cSVA is widely used in the evaluation of cervical spine diseases and orthopedic surgery. A large number of studies have reported the relationship between cervical spine balance parameters and health-related quality of life (HRQOL) [4–6]. But, it mainly focuses on the prognosis of patients and postoperative cervical alignment. Few studies have systematically evaluated the relationship between preoperative cervical sagittal parameters and cervical symptoms in patients with degenerative cervical kyphosis. T1S has always been considered as an effective index to connect the cervico-thoracic spine. There is a clear correlation between T1S and C2–C7 angle, cSVA. T1S is an important parameter to evaluate the sagittal alignment of the cervical spine and has the effect of predicting postoperative cervical curvature changes [7, 8]. Studies have shown that C7, as the adjacent vertebral body of T1, is highly similar in vertebral structure and biomechanics [9]. Furthermore, initially studies have confirmed the compensatory relationship between sagittal parameters of upper and lower cervical segments [10, 11]. Notably, in these studies, the consistency of the craniocervical and cervical-thoracic structure is split, which is bound to affect the results of the study. As a new index, C2S has a unique form of expression. It is located in the upper cervical segment and participates in the craniocervical movement. At the same time, it is found that there is a high degree of consistency between C2S and T1S-CL in geometric mechanics and biomorphology. Some researchers even directly regard the measured value of C2S as T1S-CL [12].

However, when cervical degenerative kyphosis occurs, there is no clear report on the changes between the cervical sagittal parameters and whether they have an impact on the craniocervical system. In addition, the movement of the cervical spine is closely related to its structure. When the structural sequence of the cervical spine changes reversely, it often leads to the abnormal distribution of cervical biomechanics, which accelerates the degeneration while affecting the cervical function. Based on the above research status, we analyzed the clinical and radiological data of 127 patients with DCS to (1) systematically describe the imaging parameters and clinical outcomes of DCS patients. (2) Explore the importance of C2S in clinical evaluation of DCK, and try to determine the threshold of C2S in combination with established radiological parameters and health quality life measurement results, so as to enhance clinicians ' understanding of DCK.

## Methods

### Patient Selection and Data Collection

Following Institutional Review Board approval, patients with adult DCS from January 2019 to July 2022 were retrospectively surveyed at a single medical centre that specialize in treating spinal deformity. The inclusion criteria were: (1) Cervical axial symptoms (neck pain, movement limitation, etc.) with or without spinal cord neurological symptoms. (2) Imaging showed cervical degenerative changes (X-ray showed osteophyte formation, intervertebral space stenosis, intervertebral joint hyperplasia, or MRI showed intervertebral disc signal changes, lamina Modic changes, ossification of posterior longitudinal ligament, etc.) [13]. Patients with trauma, tumor, infection, thoracic and lumbar pathology, other cervical diseases (such as dropped head syndrome, ankylosing spondylitis), or prior cervical surgery were excluded. Patients were categorized into two groups and compared based on C2-7 angle (C2-7 ≥ 5° as kyphosis group, C2-7 < 5° as non-kyphosis group). Demographic information of admission was recorded. All patients were evaluated using the Numeric Rating Scale (NRS), Neck Disability Index (NDI), Euroqol 5 Dimension (EQ5D). The EQ-5D profile was converted into a corresponding list values for analysis [14]. All patient-reported clinical outcomes were collected in the centre.

### Radiographic measurements

All patients underwent anteroposterior and lateral X-ray film of the cervical spine, and the imaging parameters were measured by the Picture achieve and communication system (PACS). Two independent observers (spine fellows, CZH. and LHT.) measured the param eters, and each observer repeated the measurements with 2-week intervals. The length and angle were accurate to 0.01 cm or 0.1°.

**Fig. 1:**
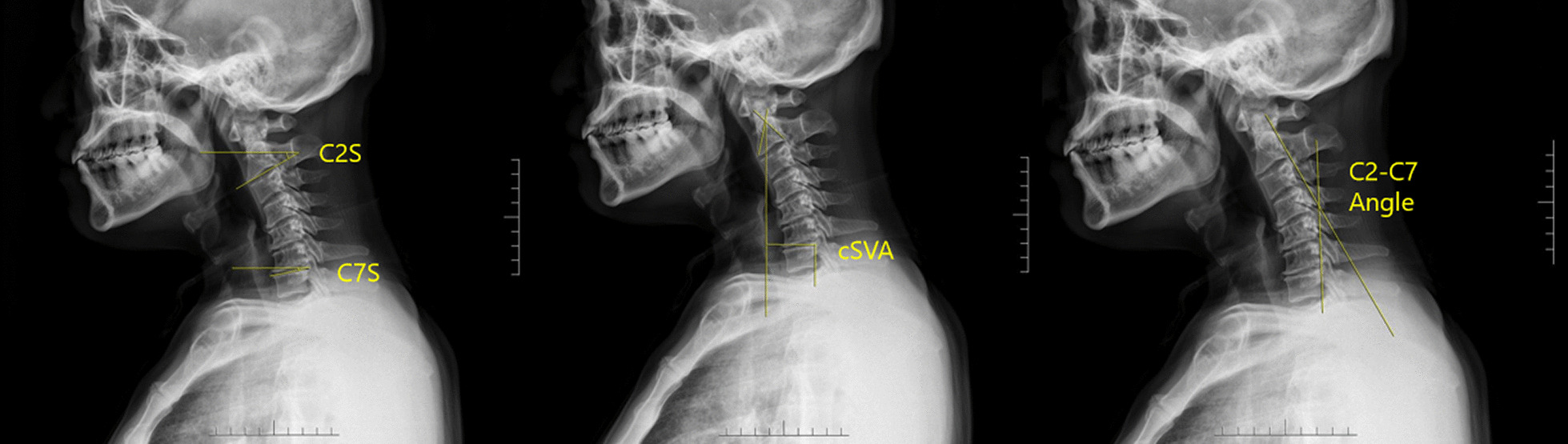
Various radiographic measurements used in this study: C2S, C7S, cSVA, and C2–C7 Angle

- O-C2 angle (Occiput-C2 angle): the angle between McGregor line (the line passing through the hard palate and the line at the end of the middle line of the occipital bone) and the endplate tangent under C2. C2–C7 angle (Jacksons method): the angle subtended by a line drawn parallel to the posterior border of C-2 and a line drawn parallel to the posterior border of C-7 [15].
- C2S (C2 slope): the angle between the lower end plate of C2 and the horizontal plane [16].
- C7S (C7 slope): the angle between the uper end plate of C7 and the horizontal plane.
- cSVA (C2-C7 Sagittal vertical axis): the horizontal distance from the plumb line of the geometric center of the C2 vertebral body to the posterior angle of the upper end plate of C7 (Fig. 1).
- T1S (T1 slope): the angle between the uper end plate of T1 and the horizontal plane.
- TS-CL: T1 slope minus cervical lordosis [17].

### Statistical analysis

SPSS 22.0 (SPSS, USA) statistical software was used to analyze the measurement results, and descriptive analysis was performed on age and gender. Measurement data were compared by independent-sample *t* test; count data were compared by *X*^2^ test. Shapiro–Wilk normality test is performed on continuous variables, which is represented as means ± standard deviations. Intra- and interobserver reliability of radiographic parameters were assessed using the intraclass correlation coefficient. Pearson correlation or Spearman correlation was used to analyze correlations between C2S and conventional radiographic parameters and clinical outcomes. Receiver operating characteristic (ROC) curve analysis was performed, and the Youden index was calculated to determine the matching C2S cutoff values according to the established radiographic parameters and clinical outcome predictors. P value less than 0.05 was considered significant.

## Results

**Fig. 2:**
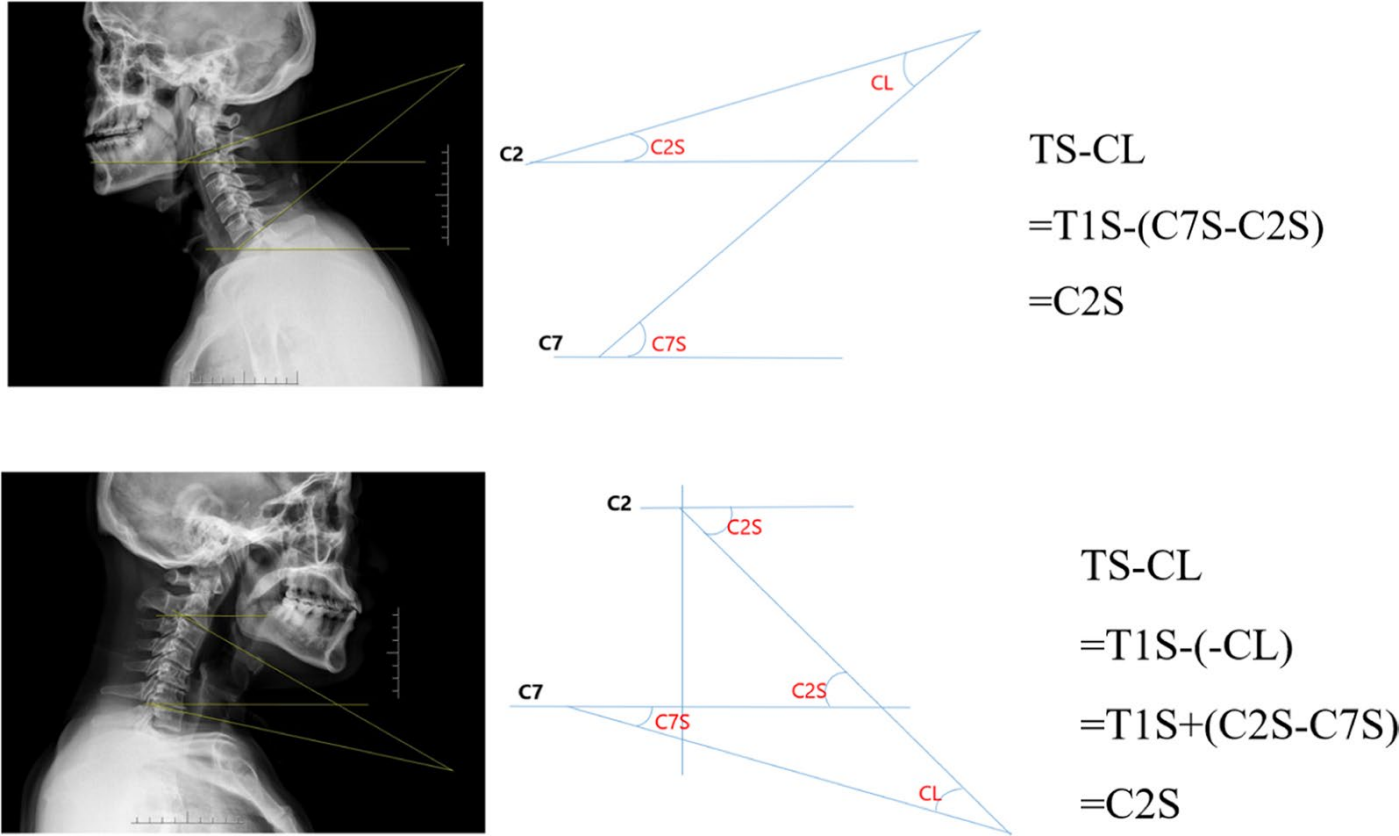
Mathematical description of C2 slope with different cervical morphologies

There were 127 patients who met inclusion criteria (79 men and 48 women). Average 56.00 ± 10.27 years old (range 31–81 years old) was divided into two sagittal angle groups based on standard neutral lateral radiographs (kyphosis = 62, non-kyphosis = 65). The average and standard deviation of the investigated radiographic parameters and clinical outcomes are provided in Table 1. The measured values of C2S and TS-CL are almost identical. In addition, we perform mathematical verification by geometric transformation (Fig. 2). The values of C2S in the two sagittal plane angle groups are quite different, suggesting that C2S plays a role in the change of kyphosis. Aggravating cervical kyphosis increases cSVA positively. Of the 127 cSVA measurements, there was only one patient with a plumb line posterior to the posterior aspect of C7 (‘‘negative sagittal balance’’). Good-to-excellent intra- and interobserver reliability for all radiographic measurements was observed. NRS, NDI and EQ5D in the kyphosis group were more severe than those in the non-kyphosis group, indicating that changes in cervical morphology can affect pain and disability.

**Table 1:** Descriptive information on radiographic parameters and clinical outcomes of patients

Parameter	Kyphosis group				Non-kyphosis group			
	Total (*n* = 62)	Female (*n* = 25)	Male (*n* = 37)	*p* value	Total (*n* = 65)	Female (*n* = 23)	Male (*n* = 42)	*p* value
C0–C2 angle (°)	− 24.78 ± 5.88^b^	− 24.13 ± 5.47	− 25.22 ± 6.18	0.480	− 20.98 ± 9.38^b^	− 19.19 ± 7.49	− 21.95 ± 10.22	0.258
C2–C7 angle (°)	9.95 ± 3.16^b^	9.82 ± 3.35	10.04 ± 3.06	0.786	− 17.27 ± 8.12^b^	− 18.73 ± 7.70	− 16.47 ± 8.32	0.288
C2 slope (°)	25.82 ± 3.89^b^	24.76 ± 3.32	26.54 ± 4.12	0.078	11.97 ± 4.72^b^	11.12 ± 4.77	12.44 ± 4.68	0.284
C7 slope (°)	16.23 ± 4.80^b^	15.52 ± 4.27	16.71 ± 5.12	0.344	24.25 ± 7.77^b^	24.42 ± 7.12	24.16 ± 8.78	0.901
cSVA (mm)	2.66 ± 1.16^a^	2.49 ± 0.99	2.78 ± 1.26	0.343	2.19 ± 1.19^a^	1.89 ± 1.20	2.35 ± 1.17	0.139
T1 slope (°)	17.00 ± 4.29^b^	16.31 ± 3.99	17.46 ± 4.48	0.302	28.74 ± 8.21^b^	29.10 ± 7.20	28.54 ± 8.78	0.794
TS-CL (°)	26.95 ± 3.85^b^	26.12 ± 3.33	27.51 ± 4.12	0.168	11.47 ± 4.56^b^	10.37 ± 4.67	12.06 ± 4.45	0.155
*HRQOL parameters*								
NRS score	4.37 ± 2.06^b^	4.08 ± 2.22	4.57 ± 1.95	0.365	3.54 ± 1.73^b^	3.04 ± 1.87	3.81 ± 1.61	0.088
NDI (%)	30.97 ± 6.04^b^	31.20 ± 5.89	30.81 ± 6.21	0.806	26.49 ± 7.37^b^	24.96 ± 6.90	27.33 ± 7.56	0.216
EQ5D-VAS score	32.81 ± 8.05^b^	33.32 ± 8.46	32.46 ± 7.87	0.683	39.82 ± 10.97^b^	40.00 ± 8.40	39.71 ± 12.24	0.921

Compare the total number of two groups: “a” means that *p* < 0.05 and “b” means that *p* < 0.01

### Correlations

#### Between conventional radiographic parameters

**Fig. 3:**
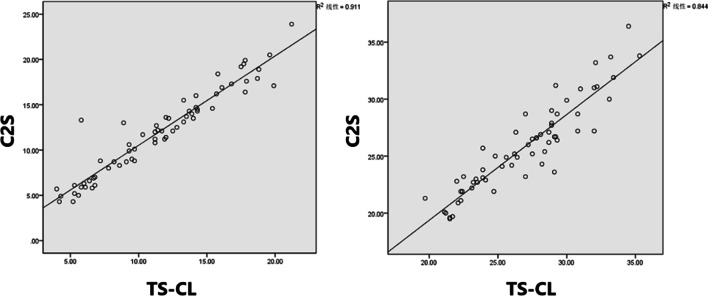
C2S was strongly linearly correlated with TS-CL on standard lateral radiographs (*R*^2^ = 0.911, non-kyphosis) (*R*^2^ = 0.844, kyphosis)

C2-C7 angle correlated with O-C2 angle (*r* =  − 0.283, *p* = 0.026 kyphosis; and *r* =  − 0.312, *p* = 0.012 non-kyphosis), C7S (*r* =  − 0.495, *p* = 0.000 kyphosis; and *r* =  − 0.795, *p* = 0.000 non-kyphosis), TS-CL (*r* =  − 0.262, *p* = 0.040 kyphosis; and *r* = 0.261, *p* = 0.036 non-kyphosis). cSVA correlated with O-C2 angle (*r* =  − 0.250, *p* = 0.049 kyphosis; and *r* = − 0.256, *p* = 0.040 non-kyphosis), C7S (*r* = 0.256, *p* = 0.045 kyphosis; and *r* = 0.399, *p* = 0.001 non-kyphosis). C2S correlated with C2-C7 angle (*r* = 0.332, *p* = 0.008 kyphosis; and *r* = 0.285, *p* = 0.021 non-kyphosis), cSVA (*r* = 0.427, *p* = 0.001 kyphosis; and *r* = 0.549, *p* = 0.000 non-kyphosis), and T1S-CL (*r* = 0.919, *p* = 0.000 kyphosis; and *r* = 0.954, *p* = 0.000 non-kyphosis) (Table 2, Fig. 3).

**Table 2:** Correlation between radiographic parameters of different types of grouping

Variable	O-C2 angle (°)	C2–C7 angle (°)	C2 slope (°)	C7 slope (°)	cSVA (mm)	T1 slope (°)
*Kyphosis group*						
C2–C7 angle (°)	*R* = − 0.283^a^	–	–	–	–	–
C2 slope (°)	*R* = − 0.467^b^	*R* = 0.332^b^	–	–	–	–
C7 slope (°)	*R* = − 0.029	*R* = − 0.495^b^	*R* = 0.451^b^	–	–	–
cSVA (mm)	*R* = − 0.250^a^	*R* = 0.193	*R* = 0.427^b^	*R* = 0.256^a^	–	–
T1 slope (°)	*R* = − 0.128	*R* = − 0.500^b^	*R* = 0.580^b^	*R* = 0.923^b^	*R* = 0.306^a^	–
TS-CL (°)	*R* = − 0.375^b^	*R* = 0.262^a^	*R* = 0.919^b^	*R* = 0.623^b^	*R* = 0.499^b^	*R* = 0.705^b^
*Non-kyphosis group*						
C2–C7 angle (°)	*R* = − 0.312^a^	–	–	–	–	–
C2 slope (°)	*R* = − 0.664^b^	*R* = 0.285^a^	–	–	–	–
C7 slope (°)	*R* = − 0.050	*R* = − 0.795^b^	*R* = 0.257^a^	–	–	–
cSVA (mm)	*R* = − 0.256^a^	*R* = − 0.142	*R* = 0.549^b^	*R* = 0.399^b^	–	–
T1 slope (°)	*R* = − 0.067	*R* = − 0.844^b^	*R* = 0.249^a^	*R* = 0.951^b^	*R* = 0.448^b^	–
TS-CL (°)	*R* = − 0.675^b^	*R* = 0.261^a^	*R* = 0.954^b^	*R* = 0.295^a^	*R* = 0.554^b^	*R* = 0.298^a^

“a” means that *p* < 0.05 and “b” means that *p* < 0.01

#### Between radiographic parameters and clinical outcome measures

In patients with kyphosis group, C2S positively correlated with NRS (*r* = 0.345, *p* = 0.006) and NDI (*r* = 0.357, *p* = 0.004). C2S negatively correlated with EQ5D-VAS (*r* =  − 0.333, *p* = 0.008). TS-CL also showed similar correlations with NRS (*r* = 0.356, *p* = 0.005), NDI (*r* = 0.382, *p* = 0.002), and EQ5D-VAS (*r* =  − 0.347, *p* = 0.006). The cSVA significantly correlated with NRS (*r* = 0.334, *p* = 0.008), NDI (*r* = 0.546, *p* = 0.000) and EQ5D-VAS (*r* = − 0.367, *p* = 0.003). O-C2 angle and C2-C7 angle were closely related to NRS (*r* = − 0.288,* p* = 0.023) (*r* = 0.756,* p* = 0.000) and NDI (*r* = − 0.287,* p* = 0.024) (*r* = 0.374,* p* = 0.003). Notably, in patients with non-kyphosis group, the relationship between HRQOL and O-C2 angle and C2-C7 angle changed, and even some correlations were lost, such as NRS and O-C2 angle, NDI and C2-C7 angle. However, data in the present study demonstrated that the relationship between C2S and HRQOL is relatively fixed, suggesting that C2S has excellent stability in evaluating the clinical results of DCS (Table 3).

**Table 3:** Correlation between radiographic parameters and clinical outcome measures of different types of grouping

Parameter	O-C2 angle (°)		C2–C7 angle (°)		C2 slope (°)		C7 slope (°)		cSVA (mm)		T1 slope (°)		TS-CL (°)	
	*R*	*P*	*R*	*P*	*R*	*P*	*R*	*P*	*R*	*P*	*R*	*P*	*R*	*P*
*Kyphosis group*														
NRS	− 0.288	0.023	0.756	0.000	0.345	0.006	− 0.261	0.040	0.334	0.008	− 0.236	0.064	0.356	0.005
NDI	− 0.287	0.024	0.374	0.003	0.357	0.004	0.026	0.843	0.546	0.000	0.068	0.601	0.382	0.002
EQ5D-VAS	0.240	0.060	− 0.710	0.000	− 0.333	0.008	0.258	0.043	− 0.367	0.003	0.211	0.099	− 0.347	0.006
EQ5D-TTO (Spearman)	0.210	0.102	− 0.700	0.000	− 0.300	0.018	0.153	0.236	− 0.489	0.000	0.134	0.299	− 0.375	0.003
*Non-kyphosis group*														
NRS	− 0.223	0.074	0.405	0.001	0.358	0.003	− 0.176	0.160	0.473	0.000	− 0.181	0.149	0.395	0.001
NDI	− 0.252	0.043	− 0.080	0.526	0.599	0.000	0.388	0.001	0.862	0.000	0.410	0.001	0.595	0.000
EQ5D-VAS	0.129	0.305	0.000	0.999	− 0.434	0.000	− 0.227	0.069	− 0.712	0.000	− 0.241	0.053	− 0.433	0.000
EQ5D-TTO (Spearman)	0.242	0.052	− 0.302	0.015	− 0.231	0.064	0.130	0.301	− 0.477	0.000	0.126	0.319	− 0.260	0.037

### C2S cutoff values as a potential predictor of cervical sagittal alignment

**Fig. 4:**
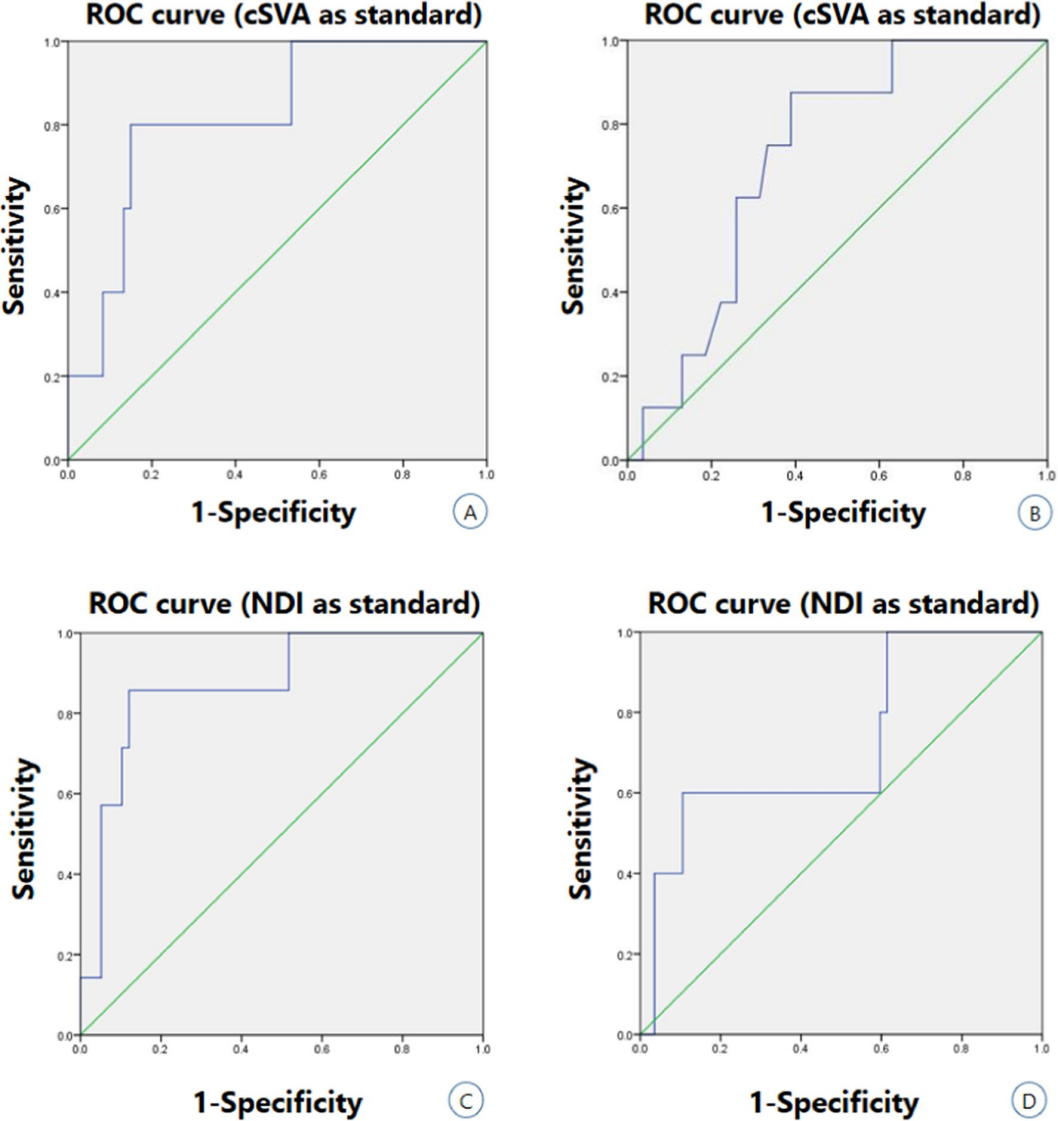
ROC analysis to determine matching C2S cutoff values basing to established radiographic parameters and clinical functional disability predictors. **A**, **B**: C2S cutoff value according to a cSVA of 40 mm (C2S 16.3°, AUC 0.820, sensitivity 0.800, specificity 0.850, non-kyphosis)/(C2S 26.3°, AUC 0.721, sensitivity 0.875, specificity 0.611, kyphosis). **C**, **D**: C2S cutoff value according to severe disability by NDI (C2S 16.3°, AUC 0.872, sensitivity 0.857, specificity 0.879, non-kyphosis)/(C2S 30.5°, AUC 0.723, sensitivity 0.600, specificity 0.895, kyphosis)

For the subgroup of patients presenting with DCK, C2S had cutoff points of 26.3° (Fig. 4B) and 30.5° (Fig. 4D) according to a cSVA of 40 mm (area under the curve [AUC] 0.721, sensitivity 0.875, specificity 0.611), and severe disability expressed by NDI (NDI ≥ 40%; AUC 0.723, sensitivity 0.600, specificity 0.895). However, for other subgroups of patients, C2S had cutoff points of 16.3° (Fig. 4A) and 16.3° (Fig. 4C) according to a cSVA of 40 mm (AUC 0.820, sensitivity 0.800, specificity 0.850), and severe disability expressed by NDI (NDI ≥ 40%; AUC 0.872, sensitivity 0.857, specificity 0.879) (Fig. 4).

## Discussion

Previous studies have reported the relationship between cervical sagittal parameters, such as the negative correlation between upper cervical spine (O-C2 angle) and lower cervical spine (C2-C7 angle), the influence of T1S changes on C2-C7 angle and the clinical application of cSVA in evaluating cervical balance [18, 19]. However, as the most dexterous segment of the whole spine, the cervical spine undertakes the skull and maintains visual stability. While ensuring the upright walking of the human body, it often bears a large axial compression load. Because the physiological stress distribution of the cervical spine is different from that of the lumbar spine, it mainly depends on the double posterior column to share the strength, which leads to the cervical spine that is more prone to degeneration. When degenerative kyphosis occurs in the cervical spine, there is no clear conclusion on how the relationship between sagittal parameters changes and whether the commonly used indicators will mismatch. Therefore, by comparing the differences in clinical symptoms and radiological parameters between DCK patients and N-DCK patients, this study can systematically describe the cervical sagittal parameter relationship of DCK patients, to more accurately evaluate the structural characteristics of DCK.

As a balance index reflecting the relationship between the center of gravity of skull and the sagittal position of spine, cSVA has profound clinical significance in evaluating neck balance. Changes in cervical curvature have long been considered a key factor affecting cervical sagittal stability; Yang et al. believe that cervical sagittal balance often depends on the mutual restraint between the upper and lower cervical spine [20]. Wang et al. found that changes in head position first affect the stability of the O-C2 segment due to its greater flexibility and compensatory capacity [21]. Some authors have studied that CL has no correlation with cSVA [22]. Based on the study of degenerative cervical disease, Weng et al. found that O-C2 angle is closely related to cSVA, while C2-7 angle and cSVA are not clear [23]. So are our results. We also found that the aggravation of cervical kyphosis will be accompanied by the forward movement of the skull, and cSVA will increase positively. A systematic review suggests that severe sagittal imbalance occurs when cSVA is greater than 40 mm, and increased cSVA was shown to correlate with an increased cross-sectional surface area of the cervical foramina [24]. An interaction was noted between alignment and cSVA such that increasing cSVA in patients with cervical kyphosis may be associated with higher cord signal intensity. This is similar to the study of Virk et al. They believed that with the increase of cSVA, patients with kyphosis measured by mJOA and NDI scores showed more obvious severity of myelopathy [25]. A biomechanical study of an in vitro experimental model showed that the increase in cSVA was related to the shortening of the occipital extensor and the extension of the cervical extensor, which corresponded to the flexion of C2-C7 and the extension of O-C2 [26]. Our study found that NRS score and NDI were closely related to O-C2 angle (*r* = − 0.288,* p* = 0.023) (*r* = − 0.287,* p* = 0.024), C2-C7 angle (*r* = 0.756,* p* = 0.000) (*r* = 0.374,* p* = 0.003) and cSVA (*r* = 0.334,* p* = 0.008) (*r* = 0.546,* p* = 0.000) in DCK patients, indicating that neck muscles bear a greater load during the increase in cSVA positivity, which may be the main cause of headache and neck axial symptoms. Clinically, many patients with degenerative cervical kyphosis are often associated with cervical spondylotic myelopathy. From the patho-physiological and morphological aspects, it is because the changes in the structure of cervical kyphosis reduce the space around the spinal cord and nerves, resulting in static compression and nerve injury. We speculate that this change will gradually occur and have an effect on the overall cervical spine.

In addition to the local interaction of the cervical spine, changes in the thoracic spine also affect cervical curvature. Studies by Hofler and colleagues suggest that any increase in T1S must require compensatory increases in cervical lordosis in order to maintain horizontal gaze [27]. Some studies related to recurrent kyphosis after cervical spine surgery have shown that T1S can predict the development of cervical kyphosis, and they believe that T1S > 40° is associated with worse EQ5D health status scores [28, 29]. However, in this study, only 1/5 of the patients could clearly observe the upper edge of the T1 vertebra, while 4/5 of the patients observed the C7 vertebra. A comparative study showed that highly similarity between C7S and T1S, but T1S was only visible in 18% of the population, whereas C7S accounted for 82% [30]. Núñez-Pereira et al. reported that there was a certain correlation between C7S and O-C2 angle, C2–C7 angle, they found that the smaller the C7S forward, the worse the neck compensatory ability [31]. In a prospective study, Le Huec et al. found that C7S increased with the increase of cervical lordosis, but O-C2 angle was not affected by C7S changes, which was consistent with this study [32]. This study found that C7S had a strong correlation with C2–C7 angle and cSVA (*p* < 0.01). Importantly, this correlation was less affected by cervical morphology. This may explain why C7S is of great significance in the evaluation of clinical improvement and sagittal balance after cervical spine surgery. Therefore, we believe that C7S can effectively replace T1S.

In previous studies of the spine-pelvis in adult sagittal deformity, the morphological PI of the pelvis has been shown to predict an ideal lumbar kyphosis (LL), but there is no same guiding parameter in the cervical spine [33]. TS-CL is a composite index that better describes the harmony between cervical alignment and thoraco-lumbar alignment on the basis of T1S and cervical lordosis. Studies have showed that the mismatch between the TS-CL exceeds 17°, then cervical deformity is present [34]. In patients undergoing cervical fusion, Sharma et al. found a strong correlation between TS-CL and cSVA. They showed that the mismatch of TS-CL over 20° was associated with cSVA over 4 cm [35]. However, TS-CL cannot be obtained directly from the standard neutral lateral radiographs. It needs to be measured three times and then superimposed. This measurement error will greatly reduce the reliability of the experimental results. Protopsaltis et al. summarized the concept of cervical-thoracic matching and cervical landmarks into a simple slope measurement by comparing the sagittal parameters of patients with cervical and cervico-thoracic deformities [36]. As an effective index for connecting the cranio-cervical and evaluating the spine-pelvis, C2S may have similar predictive value as cSVA in the preoperative planning of cervical spine orthopedic reconstruction. Since the measured values of C2S and TS-CL are similar and the strongest correlation between them is observed, we can quickly derive the relationship between TS-CL and C2S by geometric transformation (Fig. 2). This transformation makes clinical discussion and research analysis easier. Notably, compared with TS-CL, C2S retains the continuity of cranio-cervical and cervico-thoracic structure. C2S is a structural index in the upper cervical region, which can directly reflect the alignment of local segments. At the same time, we can indirectly reflect the matching degree of cervical and upper thoracic by observing the compensation of C2S connection alignment to cervical lordosis. When CL is not enough to meet T1S, the upper cervical spine will hyperlordosis, increasing the angle of C2S. In addition, the statistical association between cSVA with C2S and C7S may confirm our hypothesis that the effects of this degenerative deformity can extend to longer segments and even disrupt spinal regional balance. This is similar to the conclusion of Ramchandran et al. [37]. As a consequence, this may require early active clinical intervention. Understanding the compensatory mechanism associated with this degenerative kyphosis can not only comprehensively describe the characteristics of this deformity, but also help to avoid excessive fusion to achieve the best surgical results.

Kim et al. conducted a mid-term follow-up of 111 patients who underwent multilevel cervical fusion and found that C2S was potentially useful in predicting postoperative clinical outcomes in patients with multilevel fusion [38]. He believed that patients with cervical malalignment were more prone to fatigue in cervical muscle tissue, the compensatory mechanism of maintaining upright posture and visual homeostasis would lead to a decrease in HRQOL. In the present study, C2S showed moderate or above correlation with HRQOL-related metrics investigated including the NRS, NDI and EQ5D-VAS. The C2S in the kyphosis group was greater than that in the non-kyphosis group. We believe that the forward tilt of the C2 vertebra compensates for the forward movement of the skull center of gravity and the non-uniform compression of the intervertebral disc caused by DCK, but this morphological change makes the gravity load distribute in opposite directions. Since the limited bearing capacity of the bone-joint-ligament structure, in order to maintain the alignment of the cranio-cervical, the work of the cervical extensor group is increased, which makes the muscles continuously tense. This may explain the contribution of C2S in neck pain and disability.

At present, there are few reports on the “normal” range of C2S, especially when the degenerative changes of the cervical spine are unclear. Previous studies reported TS-CL values of about 13.9° to 16.5° in normal adult cohorts [39, 40]. Due to the strong similarity between C2S and TS-CL, many authors choose direct equivalent substitution. We determined matching C2S cutoff values based on established imaging parameters and clinical outcome predictors to make the results clearer and more reliable. ROC curve analysis in this study showed that the cut-off value of C2S in the kyphosis group matched with the baseline cSVA of 40 mm was 26.3°, and the cutoff value of C2S in the lordosis group was 16.3°. Severe disability expressed by NDI matched a C2S of 16.3° in kyphosis group and C2S of 30.5° in lordosis group. This result is significantly higher than the cut-off value of C2S reported by Kim et al. (cSVA:18.8°, NDI:22.25°) [38]. This difference may be caused by different patient cohorts. Their study cohorts were mainly patients with cervical spondylotic myelopathy and were not grouped according to cervical morphology. Notably, our study cohort was patients with degenerative cervical spondylosis. Considering the long-term strain of cervical muscle tissue and the decrease of the overall compensatory ability of the spine, it may be understood why the results of this study are smaller than the C2S cutoff value reported by Protopsaltis et al. based on adult cervical deformity [36].

In summary, this study is a single-center retrospective analysis, mainly on the DCK population cervical sagittal parameters and the correlation between the parameters was systematically described, we emphasized the C2S in clinical evaluation and preoperative planning of the importance and practicality. In our research, there are some limitations: First, the number of cases of DCK is relatively small, and there is no multicenter sample control, which may have no response bias. In the future, larger prospective studies are needed to further validate C2S and other sagittal alignment parameters as predictors of clinical outcomes. Second, this study only described the relationship between the radiological parameters of the sagittal plane of the DCK cervical spine and did not correlate the dynamic X-ray and MRI results. Future work should further explore the relationship between degenerative cervical kyphosis and spinal cord morphology, so as to help clinicians deeply understand the pathophyiological progress of cervical spondylotic myelopathy with kyphosis. Thirdly, the cranio-cervical, spine-pelvic parameters and thoracic alignment form a correlation chain. The cervical curvature and morphological changes are not simply local problems, but also need to be considered at the whole spine level. As a consequence, we need longitudinal data to study the relationship between cervical spine and spine-pelvic parameters.

## Conclusion

In this study, the cervical sagittal parameters of DCK population and the correlation between the parameters were systematically described. When cervical degenerative changes, we first proposed the trend of cervical sagittal alignment and the cutoff value of C2S in different cervical curvature. Data in the present study demonstrated the correlation between C2S with cervical sagittal alignment and HRQOL, indicating the importance and practicability of C2S in clinical evaluation and preoperative planning.
